# Establishing the interchangeability of arterial stiffness but not endothelial function parameters in healthy individuals

**DOI:** 10.1186/s12872-019-1167-3

**Published:** 2019-08-06

**Authors:** Raissa Perrault, Alexander Omelchenko, Carla G. Taylor, Peter Zahradka

**Affiliations:** 1 Canadian Centre for Agri-Food Research in Health and Medicine, St. Boniface Albrechtsen Research Centre, 351 Tache Avenue, Winnipeg, MB R2H 2A6 Canada; 2 Department of Physiology and Pathophysiology, Faculty of Health Sciences, University of Manitoba, Winnipeg, Canada; 3 Department of Food and Human Nutritional Sciences, Faculty of Agriculture and Food Science, University of Manitoba, Winnipeg, Canada

**Keywords:** Arterial stiffness, Endothelial dysfunction, Pulse wave velocity, Augmentation index, Reactive hyperemia

## Abstract

**Background:**

Development of instruments capable of detecting early stage vascular disease has increased interest in employing arterial stiffness (e.g. pulse wave velocity (PWV), augmentation index (AIx)) and endothelial dysfunction (e.g. reactive hyperemia index (RHI)) to diagnose atherosclerotic disease before occurrence of a cardiovascular event. However, amongst the equipment designed for this purpose, there is insufficient information regarding each of these parameters to establish appropriate cutoffs to distinguish between healthy and unhealthy blood vessels. To address these limitations, the study was designed to establish the upper arterial stiffness and endothelial function thresholds in a healthy population, by comparing the outputs from different instruments capable of measuring PWV, AIx and RHI.

**Methods:**

A systematic comparison of PWV, AIx and RHI was conducted to determine the inter-relationships between these parameters of vascular functionality. Outputs were obtained non-invasively using three instruments, the VP-1000 (VP), SphygmoCor (SC), and EndoPAT (EP), in 40 apparently healthy males and females.

**Results:**

Correlations were found between the brachial-ankle PWV and radial-ankle PWV (by VP and SC), and PWV (VP) with AIx (SC). The interchangeability of these outputs was demonstrated by the Bland Altman test, making it feasible to extrapolate cut-offs for radial-ankle PWV and AIx equivalent to brachial-ankle PWV that signify healthy vessels. In contrast, RHI showed no association with AIx, suggesting these endothelial and arterial parameters are functionally distinct.

**Conclusions:**

It was concluded that it is possible to compare the vascular function outputs of different instruments and identify healthy from unhealthy vessels, even though the approaches for quantifying the underlying physiological processes may differ. In this way, non-invasive determination of arterial function could be a new paradigm for detecting existing early stage asymptomatic atherosclerotic disease in individuals using techniques that are amenable to the clinical setting.

**Electronic supplementary material:**

The online version of this article (10.1186/s12872-019-1167-3) contains supplementary material, which is available to authorized users.

## Background

The inability to easily predict the likelihood of a cardiovascular event at the individual level [1–4] has led to interest in developing instruments capable of diagnosing early stage cardiovascular disease (CVD), before the appearance of symptoms. It is thought that arterial stiffening and endothelial dysfunction are among the earliest vascular properties altered with the onset of CVD [5, 6]. Accordingly, a number of important CVD risk factors, including hypertension, lifestyle and age, have a strong association with both arterial stiffening and endothelial dysfunction [7–12]. Arterial stiffening, or a decrease in the ability of an artery to distend due to structural changes in the components of the elastic artery walls [13–15], results in the transmission of damaging pulsatile flow through the circulatory system. This parameter can be assessed through measurement of blood flow rate (pulse wave velocity, PWV) or by analysis of the shape of the arterial pressure waveform (pulse wave analysis, PWA), which provides an augmentation index (AIx) that is based on reflection of the pulse wave from branch points in the arterial tree [16]. In endothelial dysfunction, the cells lining the artery wall become unable to respond to shear stress as a result of changes in blood flow, and this in turn affects arterial tone. As a result, it is possible to measure endothelial dysfunction via reactive hyperemia (RHI), which measures arterial dilatation in response to a brief period of ischemia [17].

At this time, the most widely accepted (i.e. gold-standard) method of determining arterial stiffness is carotid-femoral (cf-) PWV [18] for which reference and normal values have been published [19], although they have not yet been accepted for clinical diagnosis. However, there are a number of theoretical and practical issues that make use of this method challenging [20], including sensitivity issues associated with applying probes to the groin region as well as concerns with manipulating the carotid of persons with extensive plaque. With respect to endothelial dysfunction, the most effective method is flow-mediated dilatation (FMD), which employs ultrasound to determine the change in the dimension of the brachial artery lumen in response to ischemia [21]. While the correlation of FMD with endothelial dysfunction is excellent, the technical skills required to minimize variability for this procedure have kept it from being routinely used [22].

A number of alternative non-invasive approaches for measuring arterial stiffness and endothelial dysfunction are now available, and there is evidence to show they are able to discriminate between populations that are healthy from those with cardiovascular disease [23]. For example, brachial-ankle (ba-) PWV has been replacing cf-PWV because cut-off values for the healthy range have been identified [24], while fingertip applanation tonometry offers a less subjective and easier to perform alternative to FMD for measuring RHI. As well, several different instruments can perform PWA. However, since the numerical output of the various instruments varies, comparisons of the results are challenging even though the same vascular parameters are purportedly being measured. Thus, there is an inherent limitation to using these non-invasive instruments to follow disease progression and/or the effectiveness of a specific intervention, including those employing a lifestyle or nutritional approach as an alternative to pharmaceuticals [25]. Therefore, this study was designed to determine the thresholds for PWV, AIx and RHI indicative of arterial elasticity and endothelial function characteristic of a healthy population for eventual application in both diagnosis and management.

## Methods

### Aim and study design

Our objective was to conduct a systematic comparison of the PWV, AIx and RHI outputs for several different instruments using an apparently healthy cohort and determine the correlation between these surrogates of vascular responsiveness. This information was used to extrapolate the cut-off values representative of healthy vessels for each of the parameters (PWV, AIx) from the various instruments. Our decision to use an apparently healthy population for this study was two-fold; first, to eliminate any interference created by several important CVD risk factors (e.g. circulating lipids, high blood pressure, smoking) that might alter the vascular properties of interest, and second, to avoid confounding results arising from a mix of healthy and diseased participants, as noted in previous arterial stiffness studies [26]. Forty apparently healthy, non-smoking participants were recruited from the community. The subjects ranged in age from 23 to 71 years old (mean = 39.7 ± 13.1) and were free of any clinically diagnosed disease or infection requiring medical treatment, as assessed through medical history. Subjects taking dietary supplements (e.g. vitamins, omega-3 oils) in doses above what are achievable in a balanced diet were not included in the study, while those taking acceptable levels of supplements were included only if their consumption was steady for the 3 month period before measurements were taken. Informed consent was obtained from all participants before their inclusion in the study and prior to performing any study-related procedures. All procedures were carried out at the Asper Clinical Research Institute of St. Boniface Hospital after approval of the experimental protocol, which conforms to the ethical guidelines of the 1975 Declaration of Helsinki, by the Research Ethics Board of the University of Manitoba and by the Research Review Committee of St. Boniface Hospital.

Once enrolled, participants were asked to fast for a minimum of 12 h and to refrain from strenuous exercise for a minimum of 3 h prior to their test visit. After obtaining demographic information and measuring height, weight, and waist circumference, participants were placed in a supine position in a dimly lit room and allowed to rest for a period of 10 min before measuring blood pressure (BP) with the automated BPTru oscillometric blood pressure monitor (VSM MedTech, Coquitlam, BC). All vascular parameters were assessed subsequently during the same 1-h visit and in the same order for all participants as described below.

### Vascular measurements

SphygmoCor-Px System (AtCor Medina, Sydney, Australia): (i) radial-ankle (ra-) PWV. ECG leads were placed on each wrist and lower left abdomen. Measurements were manually taken along the surface of the skin for the following: Radial to sternal notch, sternal notch to umbilicus and umbilicus to ankle (posterior tibial). Pulse waves were collected by applanation tonometry at the radial and posterial tibial arteries, ensuring that the internal quality controls were met (standard deviation (SD) < 6% mean time and PWV SD < 10%). The radial and ankle sites were selected for standardized comparison of PWV data between instruments. (ii) PWA. A signal was collected over the radial pulse in the right arm, ensuring its internal quality controls were met (pulse height ≥ 80; pulse variation ≤5; diastolic variation ≤5; operator index ≥80%). Measurements for PWV and PWA were performed in triplicate. The instrument provides the PWA output as both aortic (central) AIx and aortic AIx normalized to 75 bpm (AIx@75).

VP-1000 system (Omron Healthcare, Bannockburn, Illinois): (i) ba-PWV. Pressure cuffs were placed around each upper arm and ankle, and a phonocardiogram (PCG) sensor was placed over the left edge of the 4th rib to detect heart sound. ECG electrodes were placed on the inside of each wrist. This assessment was performed twice and PWV was determined by the instrument using the height of the participant to derive the distance travelled by the pulse wave.

EndoPAT 2000 system (Itamar Medical, Franklin, MA): (i) RHI and (ii) PWA. Pneumatic probes were placed on the index finger of each hand and a blood pressure cuff was placed on the upper arm of the non-dominant arm. Baseline measurements of blood flow were collected for 5 min before inflation of the cuff to 50 mmHg above systolic BP. After obtaining readings for another 5 min, the cuff was rapidly deflated and additional readings were taken for the final 5 min. Both RHI and AIx were calculated by the instrument software from the same data set.

### Statistical analysis

Statistical analyses were performed using Statistical Analysis Software (SAS v9.2 Carey, NC, USA). Outliers were defined as greater than 2 times the standard deviation. One participant was removed from the data set because their ba-PWV was more than 3 times the standard deviation from the mean and their ba-PWV values (right - 20.4 m/s, left – 21.7 m/s) were above the cut-off for a healthy person (18.3 m/s) [24]. The Shapiro-Wilk test for normality was used to determine the distribution of the various parameters. Pearson’s product moment or Spearman’s rank correlational analyses were performed on indicated parameters, according to normality of distribution, with significance set at *p* ≤ 0.05. Agreement between the instruments measuring similar parameters was determined using a Bland-Altman representation, prepared with GraphPad 5 (Prism - La Jolla, CA, USA); agreement was accepted at bias ±2SD, where SD represents the standard deviation of the mean of the differences between instruments. To determine the threshold values, the best fit line for correlation between two parameters was generated using Origin 8.1 (OriginLab Corporation). The unknown threshold values for ra-PWV and AIx@75 (both SphygmoCor and EndoPat) were calculated relative to the published threshold for ba-PWV (18 m/s) [24] based on the equation of the line. The range for the thresholds was also obtained from the equation of the line substituting value of the slope plus and minus the standard error of the mean for the slope. Data stratified by RHI were compared by t-test or Krushal-Wallis test for parametric or non-parametric distribution, respectively.

## Results

### Population characteristics

Basic cardiovascular parameters (heart rate, systolic and diastolic blood pressure (BP)), for all participants were within the normal range for each parameter (Additional file 1: Table S1). These data, as well as the medical history verifying lack of clinically diagnosed CVD or infection requiring medical treatment and non-smoking status, establish the study population was apparently healthy.

### Comparison of vascular stiffness parameters

The mean and standard deviation of the various vascular parameters measured in this study, as well as the respective range of values are shown in Additional file 1 Table S2. Correlations between these parameters are shown in Table 1. There was a strong correlation between the ba-PWV (VP-1000) and ra-PWV (SphygmoCor). Interestingly, neither ba-PWV (VP-1000) nor ra-PWV correlated with AIx (either SphygmoCor AIx or EndoPat). However, AIx (SphygmoCor) normalized to heart rate (AIx@75) was correlated with ba-PWV (VP-1000), but no correlation was seen with ra-PWV. At the same time, both AIx and AIx@75 for SphygmoCor and EndoPat correlated well.

**Table 1:** Correlations amongst vascular parameters

	VP-ba-PWV	SC-ra-PWV	SC-AIx@75	SC-AIx	EP-AIx@75	EP-AIx
VP-ba-PWV	1	0.605 (*p* < 0.0001)	0.470 (*p* = 0.001)	0.429 (NS)	0.282^a^ (NS)	0.240^a^ (NS)
SC-ra-PWV		1	0.272 (NS)	0.220 (NS)	0.245^a^ (NS)	0.164^a^ (NS)
SC - AIx@75			1	0.952 (*p* < 0.0001)	0.690^a^ (*p* < 0.0001)	0.678^a^ (*p* < 0.0001)
SC - AIx				1	0.666^a^ (*p* < 0.0001)	0.722^a^ (*p* < 0.0001)
EP - AIx@75					1	0.953^a^ (*p* < 0.0001)
EP - AIx						1

Instrument abbreviations: *VP* VP-1000, *SC* SphygmoCor, *EP* EndoPat

Measurement abbreviations: *PWV* Pulse wave velocity, *AIx* Augmentation index, *@75* Corrected to 75 bpm, *RHI* Reactive hyperemia index, *ba* Brachial-ankle, *ra* Radial-ankle

*n* = 39

^a^correlations examined using Spearman’s rank correlation coefficients

To avoid the pitfalls associated with using correlation analysis to compare a common parameter using different instruments [27, 28], we performed a Bland-Altman assessment that is specifically designed to determine whether two distinct outputs agree or are interchangeable [29]. The Bland-Altman test involves plotting the difference between the methods against their means. Both ba-PWV (VP-1000) vs ra-PWV (SphygmoCor) (Additional file 1**:** Figure S1A) and AIx@75 (SphygmoCor) vs AIx@75 (EndoPat) (Additional file 1**:** Figure S1B) agreed at 95%, thus validating the strong potential for interchangeability of these parameters when assessed by these different instruments.

### Estimation of healthy cut-offs

While the advantage of the instruments used in this study is their ability to detect changes in vascular parameters associated with preclinical disease, the critical values that determine the cut-off between healthy and diseased vessels have not been established for these parameters outside of the SphygmoCor AIx@75 and VP-1000 ba-PWV [24, 30]. Since the Bland-Altman analysis (Additional file 1 Figure S1) suggests that PWV and AIx measured using different instruments are interchangeable, we used the upper limit for the healthy range of ba-PWV (18 m/s) [24, 31] to extrapolate the cut-offs for other vascular parameters measured in this study. By inserting the ba-PWV threshold value into the equation of the line for correlations against ra-PWV and AIx@75, it was possible to determine the healthy cut-off value for each of the other parameters (Table 2). As expected, ba-PWV was extremely close when comparing the values obtained between right and left sides, and all individuals in the cohort had values below these cut-offs. While no threshold values have been described in the literature for ra-PWV and AIx@75 (EndoPat), it has been suggested that 40% is an appropriate cut-off for AIx@75 (SphygmoCor) [30]. The cut-off value for AIx@75 (SphygmoCor) calculated from our data was 27.6%; there were 4 individuals who exceeded this cut-off, but all were below the previously published value of 40%. Note that sex was not taken into account in these calculations, although there is evidence that the cut-off should be lower for men [32]. For EndoPAT AIx@75, 5 values were above the cut-off (23.6%), however, only one of these individuals would be considered to have endothelial dysfunction (RHI < 1.67). To address the issue of variability, a range for each cut-off was defined by substituting the value of the slope ± the standard error of the slope into the equation of the line (Table 2). Interestingly, the breadth of each range closely associates with the coefficient of variability for the values of a given parameter (Additional file 1**:** Table S2). This novel observation clearly establishes that the variability of the collected data is least for PWV, regardless of the instrument used, followed by AIx/AIx@75 measured with the SphygmoCor, and is most for AIx/AIx@75 measured with the EndoPat.

**Table 2:** Cut-off values relative to ba-PWV for healthy vessels

	Calculated cut-off	Calculated Range^a^
VP-ba-PWV (m/s) right	18.0	–
VP-ba-PWV (m/s) left	18.1	17.2–19.1
SC-ra-PWV (m/s)	14.8	12.1–18.8
SC-AIx@75 (%)	27.6	9.4–45.8
EP-AIx@75	23.6	−7.0 – 54.2

Instrument abbreviations: *VP* VP-1000, *SC* SphygmoCor, *EP* EndoPat

Measurement abbreviations: *PWV* Pulse wave velocity, *AIx* Augmentation index, *@75* Corrected to 75 bpm, *RHI* Reactive hyperemia index, *ba* Brachial-ankle, *ra* Radial-ankle

*n* = 39

^a^calculated based on SEM of slope, *n* = 39

### Correlation of RHI with vascular stiffness parameters

The endothelium has been suggested to act as an important regulator of arterial stiffness [33, 34], thus we examined the correlations between RHI (EndoPat) and the vascular stiffness parameters PWV and AIx@75. RHI only correlated moderately with ba-PWV (Table 3). Interestingly, stratification of the data into healthy versus dysfunctional endothelium based on RHI 1.67 as the cutoff [35], ba-PWV correlated with RHI > 1.67 (healthy) but not RHI < 1.67 (dysfunctional). In contrast, ra-PWV and AIx@75 (SphygmoCor) did not correlate with RHI under any condition. In parallel with ba-PWV, AIx@75 (EndoPat) only correlated with RHI of participants with healthy endothelial function.

**Table 3:** Correlation of RHI with vascular parameters and stratification by endothelial function

	VP-baPWV	SC-raPWV	SC-AIx @75	EP-AIx @75
EP-RHI (n = 39)	− 0.401 (*p* = 0.0114)	−0.194 (NS)	0.067 (NS)	0.266^a^ (NS)
EP-RHI < 1.67 (*n* = 6)	−0.360^§^ (*p* = 0.0395)	−0.057^a^ (NS)	− 0.038^a^ (NS)	0.383^a^ (*p* = 0.0276)
EP-RHI > 1.67 (*n* = 33)	0.261^a^ (NS)	−0.147^a^ (NS)	0.348^a^ (NS)	0.116^a^ (NS)

Instrument abbreviations: *VP* VP-1000, *SC* SphygmoCor, *EP* EndoPat

Measurement abbreviations: *PWV* Pulse wave velocity, *AIx* Augmentation index, *@75* Corrected to 75 bpm, *RHI* Reactive hyperemia index, *ba* Brachial-ankle, *ra* Radial-ankle

^a^correlations examined using Spearman’s rank correlation coefficients

The lack of interaction between RHI and vascular stiffness was examined further by comparing the means for each stiffness parameter after stratification by RHI (Table 4). The only statistically significant difference observed was with ra-PWV (Table 4), where persons with RHI ≤1.67 (endothelial dysfunction) had greater stiffness as indicated by the higher blood flow. Interestingly, ba-PWV and AIx@75 were not different in individuals with and without endothelial dysfunction.

**Table 4:** Comparison of vascular parameters in individuals without (RHI > 1.67) and with (RHI ≤ 1.67) endothelial dysfunction

	RHI > 1.67^a^	RHI ≤ 1.67^b^	*P*
VP-ba-PWV right	12.62 ± 0.37	13.91 ± 0.56	0.13^d^
VP-ba-PWV left	13.08 ± 0.39	13.95 ± 0.81	0.24^d^
SC-ra-PWV	8.31 ± 0.27	9.20 ± 0.31	0.05^d^
SC-AIx@75	10.01 ± 1.93	11.67 ± 7.57	0.84^d^
EP-AIx@75	2.77 ± 3.24	5.50 ± 11.90	0.13^c^

Instrument abbreviations: *VP* VP-1000, *SC* SphygmoCor, *EP* EndoPat

Measurement abbreviations: *PWV* Pulse wave velocity, *AIx* Augmentation index, *@75* Corrected to 75 bpm, *RHI* Reactive hyperemia index, *ba* Brachial-ankle, *ra* Radial-ankle

^a^*n* = 33; mean ± SE

^b^*n* = 6; mean ± SE

^c^analyzed using t-test

^d^analyzed using Krushal-Wallis test

### Association of vascular parameters with physiological parameters

Since vascular stiffness in known to progress with age [10, 14], we determined whether there was a relationship between the age of participants and the various vascular parameters. Table 5 shows that age, SBP and DBP were moderately (r > 0.6) correlated with ba-PWV, ra-PWV and AIx@75, but RHI and heart rate were not associated with any of the vascular parameters.

**Table 5:** Correlation of vascular stiffness parameters with physiological characteristics

	ba-PWV	ra-PWV	SC-AIx@75	EP-AIx@75	RHI
Age	0.440^a^ (*p* = 0.005)	0.320^a^ (*p* = 0.047)	0.622^a^ (*p* < 0.0001)	0.536^a^ (*p* = 0.0004)	0.115^a^ (NS)
HR	−0.005 (NS)	0.095 (NS)	−0.233 (NS)	− 0.144^§^ (NS)	−0.164 (NS)
SBP	0.605^a^ (p < 0.0001)	0.356^a^ (*p* = 0.026)	0.730^a^ (*p* < 0.0001)	0.570^a^ (*p* = 0.0002)	−0.210^a^ (NS)
DBP	0.492^a^ (*p* = 0.015)	0.422^a^ (*p* = 0.0074)	0.517^a^ (*p* = 0.0007)	0.391^a^ (*p* = 0.014)	−0.299^a^ (NS)

Instrument abbreviations: *VP* VP-1000, *SC* SphygmoCor, *EP* EndoPat

Measurement abbreviations: *HR* Heart rate, *SBP* Systolic blood pressure, *DBP* Diastolic blood pressure, *PWV* Pulse wave velocity, *AIx* Augmentation index, *@75* Corrected to 75 bpm, *RHI* Reactive hyperemia index, *ba* Brachial-ankle, *ra* Radial-ankle

^a^correlations examined using Spearman’s rank correlation coefficients

*n* = 39

## Discussion

This study supports the conclusion that PWV and AIx obtained via different non-invasive instruments have similar capability in monitoring vascular health. Specifically, ba-PWV (VP-1000) correlated with ra-PWV (SphygmoCor), and AIx/AIx@75 (SphygmoCor) correlated with AIx/AIx@75 (EndoPat). The interchangeability of the PWV and AIx outputs from the instruments used in the present study was confirmed by the Bland Altman test [27, 28]. Although the numerical values of the outputs are different, it was possible to define the cut-offs for ra-PWV and AIx@75 that signify healthy vessels by extrapolation using the previously defined cut-off value for ba-PWV [24]. Interestingly, the only cross-parameter correlation that was observed was between ba-PWV (VP-1000) and AIx@75 (SphygmoCor), while there were no correlations obtained with either ra-PWV (SphygmoCor) or AIx@75 (EndoPat). The lack of correlation between certain parameters is likely due to one of several factors: i) the physiological processes being measured (blood flow, wave reflection, vasodilation after occlusion), ii) the physical locations used for data acquisition, and iii) the proprietary algorithm used to calculate the output. In addition, RHI showed no association with AIx, suggesting the endothelial and arterial parameters being measured may be functionally distinct. The latter is supported by the observation that age and blood pressure correlate with PWV and AIx, but not RHI.

Woodman et al. [36] previously reported that AIx, central pulse pressure and stiffness index, all derived from different instruments, could provide a comparable estimate of central arterial stiffness as determined by cf-PWV. In this study, we have expanded upon the findings of Woodward et al. [36] by extending the comparison of arterial stiffness parameters to include ba-PWV and ra-PWV. This novel comparison was possible once we had established that the outputs of the various instruments used to measure these vascular parameters were not only correlated, but that PWV measured by specific equipment, as well as AIx, could be interchanged regardless of the process used to obtain these values. Similar observations were reported by Obeid et al. [37], who compared PWV values measured at various locations in the arterial tree. Overall, these findings indicate that it is feasible under certain circumstances to compare the arterial stiffness outcomes of studies employing different instruments with different output values, specifically in relation to PWV and AIx, even though evidence suggesting the contrary has been published [38].

It may be surprising that PWV/AIx and RHI do not appear to correlate since the ability of the endothelium to respond to hormones and blood flow (as measured by RHI) is a factor in the onset of arterial stiffness (as indicated by PWV and AIx) [24]. This is contrary to past investigations in diseased populations which found that RHI correlated with increased arterial stiffness [33]. However, in the present study, we did find RHI was significantly correlated with ba-PWV and EP-AIx@75 when otherwise healthy individuals with endothelial dysfunction (RHI < 1.67) [35] were considered separately. Whether the presence or absence of a correlation between endothelial function and arterial stiffness parameters is due to physiological factors and/or data handling by the instruments remains to be determined. Also, calculation of RHI includes elements associated solely with peripheral pressure while arterial stiffness calculations depend upon both peripheral and central blood pressure, with the latter being acknowledged as the more important factor in determining disease risk [39].

A physiological basis for differences in the correlation of parameters between instruments may be the fact that these different outputs are derived from distinct arterial beds. One aspect that may determine the outputs from these instruments is the physiological state under which the measurements are acquired. Specifically, PWV and SC-AIx are obtained under homeostatic conditions while EP-AIx and RHI monitors the response of the arterial bed after the application of a stress (hyperemia). It has also been recognized previously that RHI is representative of small artery responsiveness [40]. Consequently, EP-AIx may also be representative of small arteries [40, 41], with wave reflection dependent upon the geometry and arteriole number of the microvascular network architecture [18]. Similarly, ra-PWV includes the smaller vessels of lower arm while ba-PWV does not. Thus, the significance of stiffness in the different arteries, namely the aorta, the muscular arteries and those of the microcirculation, remains to be fully elucidated [42, 43]. Nonetheless, while the arterial bed (and therefore arterial size) used to acquire the data may have a direct bearing on the numerical outputs obtained with each instrument, and thus the cut-off value that separates healthy from unhealthy vessels, this study and others [37] indicate the high degree of correlation for PWV between instruments is independent of the locations used to take the measurements.

PWV is considered a gold standard approach for determining arterial stiffness [18], and this parameter was highly correlated between instruments. There was also a high degree of reliability for PWV obtained via both VP-1000 and SphygmoCor as indicated by the low coefficient of variation (Additional file 1**:** Table S2), even though the VP-1000 uses height, which is less accurate [44], while the SphymoCor uses a direct measurement of the distance to calculate velocity. In contrast to PWV, the coefficient of variation was considerably higher for AIx obtained with either the SphygmoCor or the Endo-Pat. Interestingly, the coefficient of variation for AIx increased further when it was normalized to a heart rate of 75 beats/min, particularly for the Endo-Pat. A key feature of AIx is that each instrument uses a proprietary algorithm to extrapolate central pressure using data from a peripheral arterial bed, and the latter is different for each instrument (radial artery - SphygmoCor, fingertip - Endo-Pat). The high coefficient of variation for the AIx obtained with the Endo-Pat may be the result of its dependence upon the very small vessels of the fingertip, which may not be representative of the larger resistance arteries. On the other hand, the RHI value obtained by the Endo-Pat has a low coefficient of variation, suggesting that the lack of correlation with PWV (which also has a low coefficient of variation) in otherwise healthy persons (RHI > 1.67) is not due to the variability of the data and thus likely represents a true distinction between the parameters of endothelial function and arterial stiffness.

Measurement of vascular stiffness has remained an underutilized tool in preventative medicine, due in part to the technical demands and practical issues associated with measuring FMD and cf-PWV. As a result, ba-PWV is gaining acceptance as a clinical tool in many jurisdictions since it does not require the expertise needed for measuring either FMD or cf-PWV. In particular, instruments such as the VP-1000, which determine ba-PWV by applying four automated cuffs to the limbs and a phonograph over the heart, are easier to use and the data is acquired objectively. However, even though ba-PWV is recognized as an independent predictor of cardiovascular events in individuals with asymptomatic or established CVD [45], clinically relevant cut-offs that distinguish between healthy vessels and arterial stiffness are essential to develop guidelines for clinical diagnosis. Consequently, by improving detection of early stage asymptomatic atherosclerotic disease, more effective prevention and treatment strategies can be developed.

A limitation of the majority of studies examining arterial stiffness parameters for the purpose of clinical application is the lack of information regarding changes in this parameter in relation to disease progression. Thus, longitudinal studies should be designed to determine if arterial stiffness can be used to determine whether individuals in at-risk populations will eventually have a cardiovascular event. In this way, arterial stiffness could replace the less accurate assessment of traditional CVD risk factors that are based on epidemiological studies and are not robust at the individual level. Furthermore, recent evidence suggests that arterial stiffness represents changes associated with atherosclerotic disease progression and is not a factor in end stage disease [46]. Thus, non-invasive determination of arterial function could be a new paradigm for detecting existing early stage asymptomatic atherosclerotic disease in individuals using techniques that are amenable to the clinic setting.

## Conclusion

The results from this study suggest that it is possible to compare the vascular function outputs of different instruments even though the approach for quantifying the underlying physiological processes may differ. Thus, the establishment of proper standards, along with the development of user-friendly techniques for vascular functional could result in legitimate clinical application useful for both diagnosis and disease management. Further research is also needed to understand how endothelial dysfunction and arterial stiffness assessed non-invasively reflect the atherosclerotic process in both asymptomatic and established CVD, and how these factors affect disease progression and the response to interventions. The primary advantage of employing these new diagnostic modalities rests with their utility for detecting a decline in vascular health and therefore providing a personalized medicine approach for monitoring atherosclerosis versus traditional risk factor assessments that do not measure arterial function directly.

## Additional files

Additional file 1: **Table S1.** Participant characteristics. **Table S2.** Vascular parameter values and distribution. **Figure S1.** Agreement between outputs of devices measuring the same parameters. The differences between the absolute values obtained from the different methods are plotted against their means. Bias (solid horizontal line) is defined as the mean of the differences and the limits of agreement are set at ±2SD of this mean (dashed horizontal line). Panel A: Agreement of PWV, as measured by the VP-1000 and the SphygmoCor. Panel B: Agreement of AIx@75, as measured by the SphygmoCor and the EndoPAT. (DOCX 85 kb)

## Data Availability

The datasets used and/or analyzed during the current study are available from the corresponding author on reasonable request.

## Abbreviations

AIx: Augmentation index
AIx@75: Augmentation index normalized to 75 beats per minute;
ba-PWV: Brachial-ankle pulse wave velocity
BP: Blood pressure
cf-PWV: Carotid-femoral pulse wave velocity
CVD: Cardiovascular disease
DBP: Diastolic blood pressure
FMD: Flow-mediated dilatation
PWV: Pulse wave velocity
ra-PWV: Radial-ankle pulse wave velocity
RHI: Reactive hyperemia index
SBP: Systolic blood pressure
