# Supplementary material for: Establishing the interchangeability of arterial stiffness but not endothelial function parameters in healthy individuals
Source: BMC Cardiovasc Disord. 2019 Aug 6;19:190. doi: 10.1186/s12872-019-1167-3 (PMC6685177; doi:10.1186/s12872-019-1167-3)
Supplement: Supplementary file 1 — Table S1. Participant characteristics. Table S2. Vascular parameter values and distribution. Figure S1. Agreement between outputs of devices measuring the same parameters. The differences between the absolute values obtained from the different methods are plotted against their means. Bias (solid horizontal line) is defined as the mean of the differences and the limits of agreement are set at ±2SD of this mean (dashed horizontal line). Panel A: Agreement of PWV, as measured by the VP-1000 and the SphygmoCor. Panel B: Agreement of AIx@75, as measured by the SphygmoCor and the EndoPAT. (DOCX 85 kb) [file 12872_2019_1167_MOESM1_ESM.docx]

**SUPPLEMENTAL TABLES AND FIGURES**

**Supplemental Table 1.** Participant characteristics

| Characteristic | Mean ± SD^1^ | Normal range |
| --- | --- | --- |
| Age (years) | 39.1 ± 12.8 | n/a |
| Heart rate (beats/min)^2^ | 60.7 ± 8.6 | 45 – 95^4^ |
| Systolic blood pressure (right arm) (mmHg)^2^ | 100.1 ± 12.5 | 97-120^5^ |
| Diastolic blood pressure (right arm) (mmHg)^2^ | 72.8 ± 7.7 | 57-87^5^ |

^1^ n=39 participants (24 females, 15 males)

^2^Data averaged from 3 measurements for each participant

^4,5^ Reference ranges from [45, 46], respectively.

n/a= not applicable

**Supplemental Table 2.** Vascular parameter values and distribution

| Parameter | Mean ± SE^1^ | Range | CV^2^ |
| --- | --- | --- | --- |
| VP-ba-PWV (m/s) right | 12.5 ± 1.3 | 8.7 - 16.5 | 0.14 |
| VP-ba-PWV (m/s) left | 12.9 ± 1.3 | 9.7 - 16.2 | 0.14 |
| SC-ra-PWV (m/s) | 8.3 ± 1.1 | 5.7 - 11.4 | 0.15 |
| SC-AIx (%) | 16.5 ± 3.7 | -11.3 - 43.3 | 0.83 |
| SC-AIx @ 75 bpm (%) | 9.6 ± 1.1 | -12.3 - 35.7 | 1.28 |
| EP-AIx (%) | 10.9 ± 4.6 | -22 - 69 | 1.92 |
| EP-AIx @ 75 bpm (%) | 2.1 ± 4.4 | -27 - 60 | 9.33 |
| EP-RHI | 2.1 ± 0.7 | 1.2 - 3.3 | 0.21 |

^1^n=39

^2^coefficient of variation = standard deviation/mean×100

Instrument abbreviations: VP: VP-1000; SC: SphygmoCor; EP: EndoPat

Measurement abbreviations: PWV: Pulse wave velocity; AIx: Augmentation index; @75: Corrected to 75 bpm; RHI: Reactive hyperemia index, ba: brachial-ankle, ra: radial-ankle

**Supplemental Figure Legends**

*Supplemental Figure 1:* Agreement between outputs of devices measuring the same parameters. The differences between the absolute values obtained from the different methods are plotted against their means. Bias (solid horizontal line) is defined as the mean of the differences and the limits of agreement are set at ± 2SD of this mean (dashed horizontal line). Panel A: Agreement of PWV, as measured by the VP-1000 and the SphygmoCor. Panel B: Agreement of AIx@75, as measured by the SphygmoCor and the EndoPAT.

A.

B.
